# Supplementary material for: Improving Metagenomics Classification with Kmask: Entropy-Based Masking of Low-Complexity Regions
Source: bioRxiv. 2026 Sep 23:2026.09.22.753623. Preprint. [Version 1] doi: 10.64898/2026.09.22.753623 (PMC13622204; doi:10.64898/2026.09.22.753623)
Supplement: Supplementary file 1 [file NIHPP2026.09.22.753623v1-supplement-3.pdf]

bioRxiv preprint doi: https://doi.org/10.64898/2026.09.22.753623; this version posted September 23, 2026. The copyright holder for this preprint (which was not certified by peer review) is the author/funder, who has granted bioRxiv a license to display the preprint in perpetuity. It is made available under a CC-BY 4.0 International license.

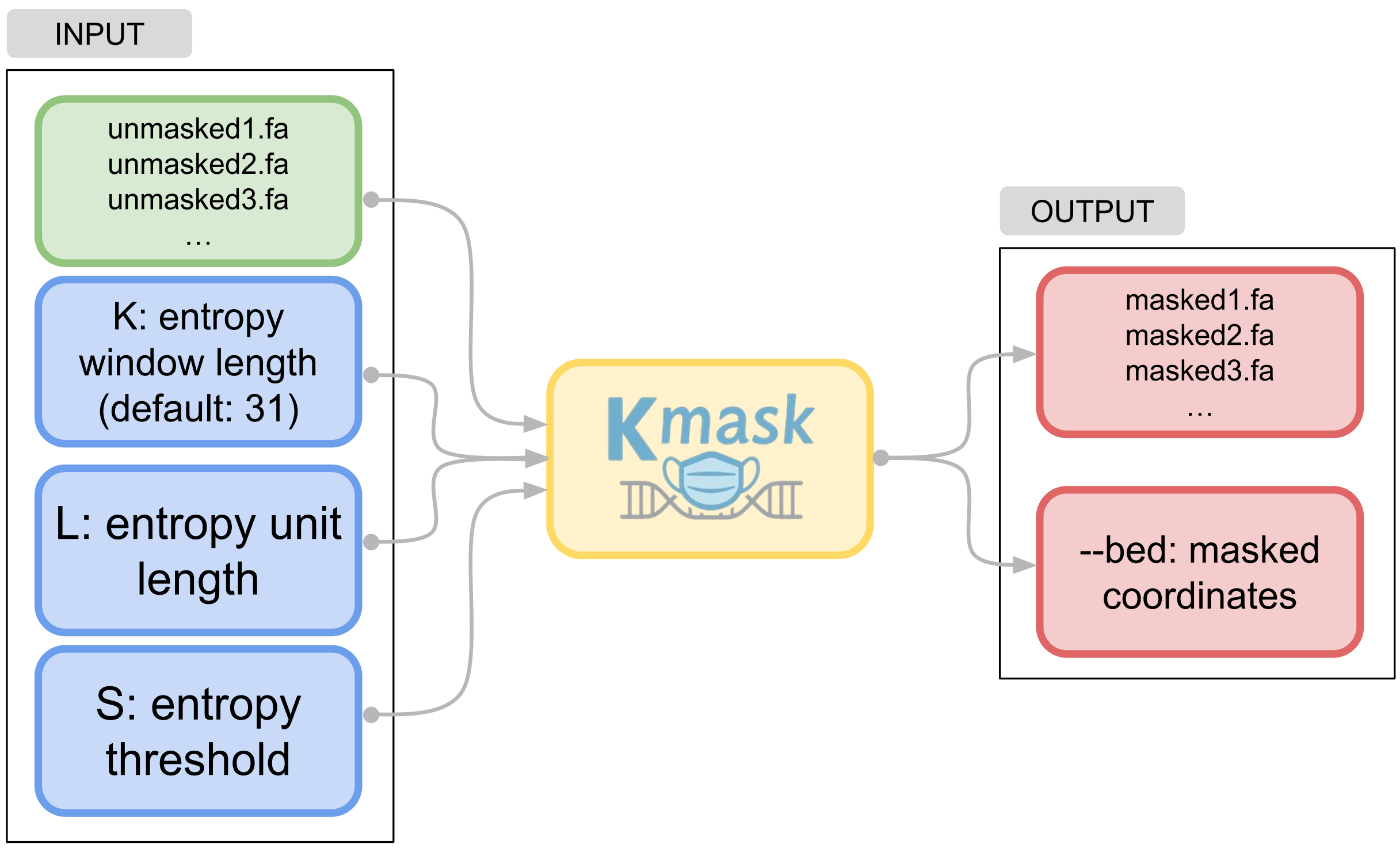

Figure S1. The Kmask processing flow. Kmask takes multiple FASTA files as input and processes them in parallel. It takes in three key parameters from the user: *k* for entropy window length (default: 31), *l* for entropy unit length (default: 3), and *s* for entropy threshold (default: 3.3). The outputs are a set of masked FASTA files, where low-entropy sequences are replaced by Ns, as well as a BED-format file specifying the coordinates of the masked regions.

bioRxiv preprint doi: https://doi.org/10.64898/2026.09.22.753623; this version posted September 23, 2026. The copyright holder for this preprint (which was not certified by peer review) is the author/funder, who has granted bioRxiv a license to display the preprint in perpetuity. It is made available under a CC-BY 4.0 International license.

Supplementary Algorithm 1. Pseudocode for the entropy calculation in Kmask.

```
Input: A string s, positive integers k and l, where k > l.
```

bioRxiv preprint doi: https://doi.org/10.64898/2026.09.22.753623; this version posted September 23, 2026. The copyright holder for this preprint (which was not certified by peer review) is the author/funder, who has granted bioRxiv a license to display the preprint in perpetuity. It is made available under a CC-BY 4.0 International license.

```
Output: A sequence (s_i, e_i) of k-mers s_i extracted from s and their respective entropies e_i.

l_in_k <- k - l + 1
q <- {s[0:l), s[1:l+1), ..., s[k - l:k)}
num_lmers = 4^l
lmer_counter <- {0, ..., 0} // length num_lmers
for lmer in q do
    lmer_counter[lmer] += 1
done
cur_s <- s[0:k)
cur_sl <- s[k - l:k)
cur_e <- ShannonEntropy(lmer_counter)
output <- ((cur_s, cur_e))

for i in [k:length(s)) do // rolling update of k-mers and Shannon entropy
    cur_s <- cur_s.pop_front().push_back(s[i]) // update k-mer
    cur_sl <- cur_sl.pop_front().push_back(s[i]) // update l-mer
    last_sl <- q.pop_front()
    q.push_back(cur_sl)
    if cur_sl != last_sl then
        last_se <- lmer_counter[last_sl] / num_lmers
        cur_se <- lmer_counter[cur_sl] / num_lmers
        lmer_counter[last_sl] <- lmer_counter[last_sl] - 1
        lmer_counter[cur_sl] <- lmer_counter[cur_sl] + 1
        new_last_sl = lmer_counter[last_sl] / num_lmers
        new_cur_sl = lmer_counter[cur_sl] / num_lmers
```

bioRxiv preprint doi: https://doi.org/10.64898/2026.09.22.753623; this version posted September 23, 2026. The copyright holder for this preprint (which was not certified by peer review) is the author/funder, who has granted bioRxiv a license to display the preprint in perpetuity. It is made available under a CC-BY 4.0 International license.

```
            // only calculate p*log2(p) for the entries that changed
            cur_e <- cur_e + last_se*log2(last_se) +
cur_se*log2(cur_se) - new_last_sl*log2(new_last_sl) -
new_cur_sl*log2(new_cur_sl)
        endif

        output.push_back((cur_s, cur_e))
done

return output
```

Supplementary Table Captions

Table S1. K-mask quality control datasets.
(a) Control genome panel. Reference-quality RefSeq genome assemblies (n = 12) spanning a range of GC content (27.0–73.0%) and taxonomic groups, used as controls for k-mer masking validation. For each genome, the table lists organism name, strain, taxonomy ID, assembly name/accession, RefSeq annotation, assembly level, contig N50, genome size, submission date, gene count, and associated BioProject/BioSample identifiers.
(b) CHM13 benchmarking. Parameter sweep evaluating kmask performance against the CHM13 human reference assembly (n = 84 parameter combinations). For each combination of k-mer size (K), a secondary parameter (L), and masking stringency threshold (S), the table reports the number of k-mer hits, false positive rate (FPR), number of k-mers masked, database size, false negative rate (FNR), true positive rate (TPR), and area under the curve (AUC).

Table S2. Reference genome assemblies used to construct Microbial2025 database.
(a–e) NCBI RefSeq/GenBank assembly summary records for archaea (n = 651), bacteria (n = 55,247), fungi (n = 66), viruses (n = 14,583), and human (n = 2) genomes included in the k-mask reference database. Each record includes assembly accession, BioProject/BioSample identifiers, organism and strain information, assembly level and status, genome and annotation submitter, release date, genome size, GC content, replicon/scaffold/contig counts, and gene counts (total, protein-coding, and non-coding).
(f) EuPathDB eukaryotic pathogen genomes retrieved from EuPathDB release 68 (n = 663), listing file name, source organism, release version, category, file contents, file format, and file size, used to extend database coverage to eukaryotic pathogens.
